# Supplementary material for: Femtosecond switching of strong light-matter interactions in microcavities with two-dimensional semiconductors
Source: Nat Commun. 2025 Jul 14;16:6490. doi: 10.1038/s41467-025-61607-2 (PMC12260094; doi:10.1038/s41467-025-61607-2)
Supplement: Supplementary file 1 — Supplementary Information [file 41467_2025_61607_MOESM1_ESM.pdf]

Femtosecond switching of strong light-matter interactions in microcavities with two-dimensional semiconductors - *Supplementary Information*

Armando Genco,[1,*] Charalambos Louca,[1,2,*] Cristina Cruciano,[1] Kok Wee Song,[3] Chiara Trovatello,[1,4] Giuseppe Di Blasio,[1] Giacomo Sansone,[5] Sam Randerson,[6] Peter Claronino,[6] Kyriacos Georgiou,[6,7] Rahul Jayaprakash,[6] Kenji Watanabe,[8] Takashi Taniguchi,[8] David G. Lidzey,[6] Oleksandr Kyriienko,[3] Stefano Dal Conte,[1] Alexander I. Tartakovskii,[6,†] and Giulio Cerullo[1,9,‡]

[1]*Dipartimento di Fisica, Politecnico di Milano, Piazza Leonardo Da Vinci 32, 20133 Milano, Italy*

[2]*NanoPhotonics Centre, Cavendish Laboratory, Department of Physics, JJ Thompson Ave, University of Cambridge, Cambridge, UK*

[3]*Department of Physics, University of Exeter, Stocker Road, EX4 4PY, Exeter, UK*

[4]*Department of Mechanical Engineering, Columbia University, New York, NY 10027, USA*

[5]*Dipartimento di Scienze Matematiche, Fisiche e Informatiche, Università di Parma, Parco Area delle Scienze 7/A, 43124 Parma, Italy*

[6]*Department of Physics and Astronomy, University of Sheffield, Hounsfield Road, S3 7RH, Sheffield, UK*

[7]*Department of Physics, University of Cyprus, 1 Panepistimiou Avenue, 2109 Aglantzia, Nicosia, Cyprus*

[8]*Advanced Materials Laboratory, National Institute for Materials Science, 1-1 Namiki, Tsukuba, 305-0044, Japan*

[9]*CNR-IFN, Piazza Leonardo da Vinci 32, Milano, 20133, Italy*

(Dated: June 23, 2025)

SUPPLEMENTARY NOTE S1: EXCITON ENERGIES AND LINEWIDTHS TRANSIENT BEHAVIOUR

In this section we present the time-dependent exciton linewidths (Fig. S1a) and line shifts (Fig. S1b) extracted from the pump-probe data taken on the MoS_2 BL excited with low fluence (Fig. 1c of the main text), using the fitting procedure used to obtain the transient exciton peak amplitudes shown in Fig. 1e. Immediately after pump excitation, both the X_A and hIX lines broaden significantly due to excitation-induced dephasing. This effect fades out in two distinct time-scales following the decay of exciton population, the first faster decay occurring below 1 ps. We observe that the first decay for hIX is faster than for X_A, the former being not pumped directly

but interacting only with the holes created in the valence band shared with X_A. Regarding the exciton line shifts, at short delay times, while the immediate blueshift of hIX can be attributed to strong repulsive Coulomb interactions, the ultrafast blueshift of X_A can be related also to the optical Stark effect [1, 2]. However, a detailed explanation of these effects in our samples goes beyond the scope of this work.

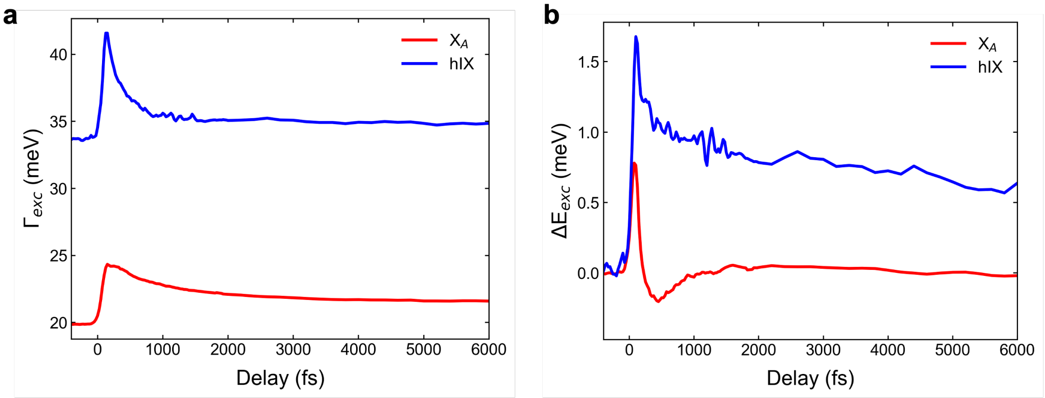

Supplementary Figure S1. a) Transient linewidths Γ_{exc} of X_A (red line) and hIX (blue line), extracted from fitting the dynamic RC in Fig.1 of the main paper. b) X_A (red line) and hIX (blue line) transient peak shifts, extracted from fitting the dynamic RC in Fig.1 of the main paper.

SUPPLEMENTARY NOTE S2: ULTRAFAST BEHAVIOUR OF MONOLAYER EXCITONS

We performed pump-probe spectroscopy experiments at low temperature (8K) on a MoS_2 ML encapsulated in hBN and placed on a DBR, to compare the excitons dynamics with the ones measured in the MoS_2 BL. For this experiment, we used broadband probe pulses and narrow band pump pulses (10 nm), the latter tuned at the energy of the intralayer A excitons ($\approx$ 1.94 eV), in a similar configuration to the measurements on MoS_2 BL shown in Fig.1 of the main text. We also pumped the ML with the same fluence used for the BL experiments (5 μJcm^{-2}). Figure S2a shows the transient reflectivity map of the ML exhibiting a strong signal at the intralayer exciton energy. This system does not support interlayer excitons being made of a single TMD monolayer.

We extracted the intralayer exciton dynamics from the transient reflectivity data following the procedure described in the main text for the BL measurements, i.e. tracing the variation of the exciton peak from the dynamic RC. Fig. S2b shows the resulting exciton population dynamics, which follow an initial ultrafast build-up, when the pump pulse excites the sample, and a double

exponential decay. The first sub-ps decay is related to bright excitons direct relaxation processes, while the second longer decay is attributed to slow exciton scattering from dark states. We observe that compared to the BL exciton dynamics, here the slow decay component is much more prominent, effectively extending the exciton population for longer times. In fact, the exciton density at 10 ps is still about 70% of the initial one. This difference compared to the BL case is probably due to the presence in the latter of fast non-radiative charge relaxation channels from K points to the energy minima of the band structure, typical of indirect bandgap semiconductors [3, 4], which limit the overall exciton lifetime.

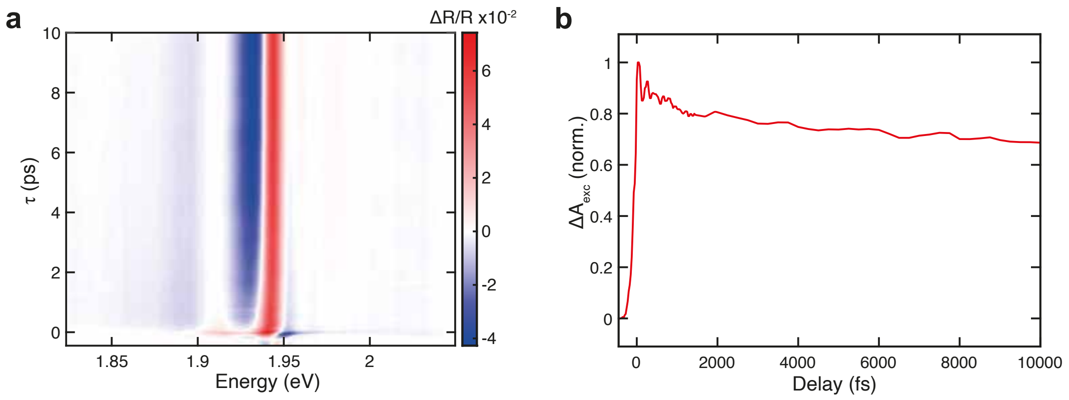

Supplementary Figure S2. a) Transient differential reflectivity color map as a function of pump-probe delay time τ and photon energy measured for MoS_2 ML. b) Normalized peak amplitude variation (ΔA_{exc}) of $X_{\mathrm{A-ML}}$, extracted from the dynamic RC at different time delays.

SUPPLEMENTARY NOTE S3: MONOLAYER CAVITY DISPERSION

Fig. S3 shows the reflectivity dispersion of a monolayer MoS_2 embedded in a microcavity. A clear anticrossing around the exciton energy is observed, resulting in lower and upper polariton branches (LPB, UPB). The RC spectra are fitted with Lorentzian functions for each angle.

A fit of the extracted peak energies with the Hamiltonian of two coupled oscillators is performed, such that $H_{\mathrm{ML}} = \begin{pmatrix} E_{\mathrm{c}} & \Omega_{\mathrm{A_{ML}}} \\ \Omega_{\mathrm{A_{ML}}} & E_{\mathrm{A_{ML}}} \end{pmatrix}$, where E_{c} and $E_{\mathrm{A_{ML}}}$ are the energies of the cavity mode and A exciton, respectively. The extracted value of Rabi splitting, $\Omega_{\mathrm{A_{ML}}}$ was 28.5 ± 0.3 meV. The A exciton energy is 1.948 ± 0.001 eV. The results of the fit are shown in Fig. S3b.

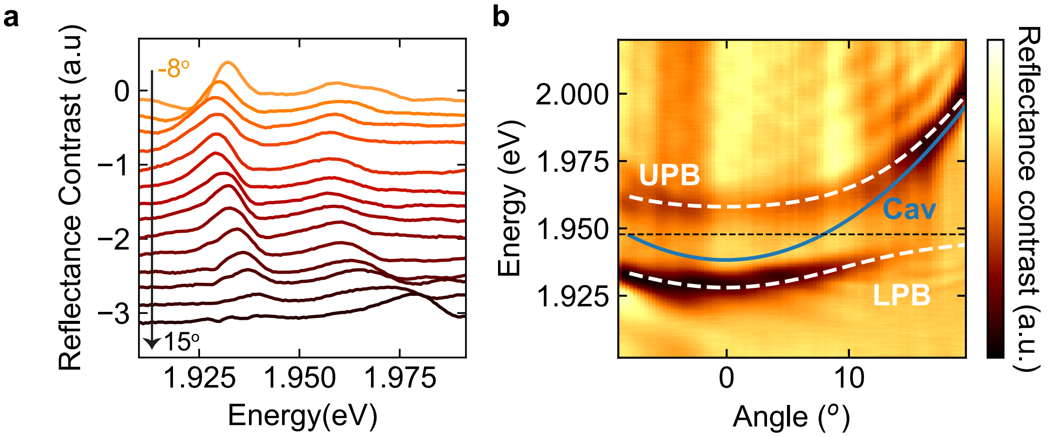

Supplementary Figure S3. a) Waterfall plot of the RC spectra as a function of angle for ML MoS_2 embedded in a microcavity. b) Energy-angle map of the cavity reflectivity spectra. The fitted upper and lower polariton branches (UPB, LPB) are shown as white dashed curves. The black horizontal dashed line corresponds to the X_A energy. The cavity mode (Cav) is shown as a blue solid curve.

SUPPLEMENTARY NOTE S4: BILAYER CAVITY TRANSIENT DIFFERENTIAL REFLECTIVITY

Fig. S4 shows the transient differential reflectivity map of the bilayer MoS_2 embedded in a microcavity, used to extract the plots in Fig.2 of the main paper, together with some spectral cross-sections taken at different time delays.

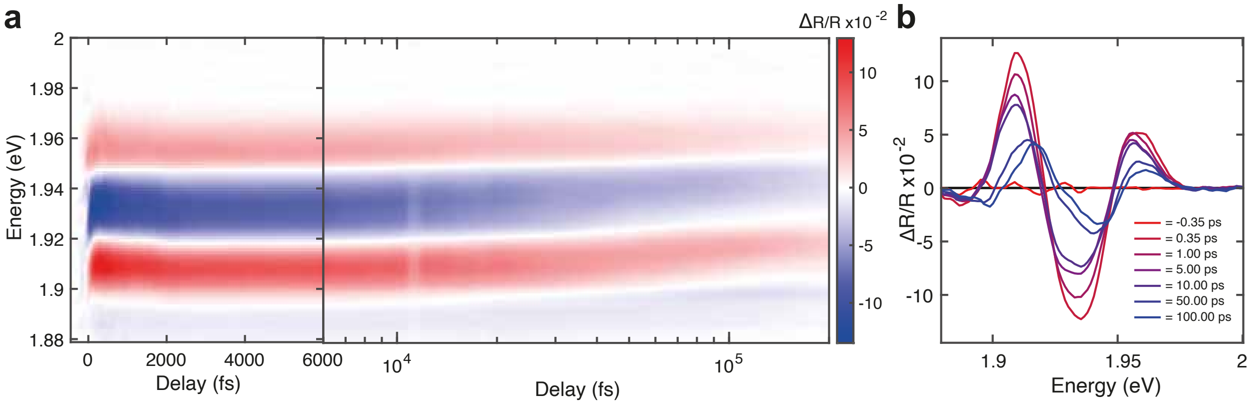

Supplementary Figure S4. a) Transient differential reflectivity color map as a function of pump-probe delay time τ and photon energy measured for the MoS_2 BL cavity. b) Spectral cross-section of the map in (a) taken at different time delays.

SUPPLEMENTARY NOTE S5: EXCITONS VERSUS POLARITONS DYNAMICS IN BL

In this section, we compare the temporal dynamics of the polariton splitting with the transient optical behaviour of the intra and interlayer excitons in the out-of-cavity sample, excited with a narrow-band pump in resonance with X_{A-BL}, using comparable pump fluences. For the cavity experiments shown in Fig. 2 of the main paper we used a fluence of 212 $\mu J/cm^2$, hence we excite the out-of-cavity sample with ∼30 $\mu J/cm^2$ in order to obtain similar excitation densities on the TMD, considering a transmittance of the top silver mirror of about 15%. The transient RC pump-probe map measured in these conditions is shown in Fig. S5. Using the same procedure based on the Transfer Matrix Method (TMM) that we present in the main text (see Methods), we fit the experimental RC spectra at each delay with Lorentzian functions to extract the dynamic lineshape of the different excitonic species. From such analysis we can isolate the contribution of the time-dependent excitonic line broadening for intralayer excitons (X_A).

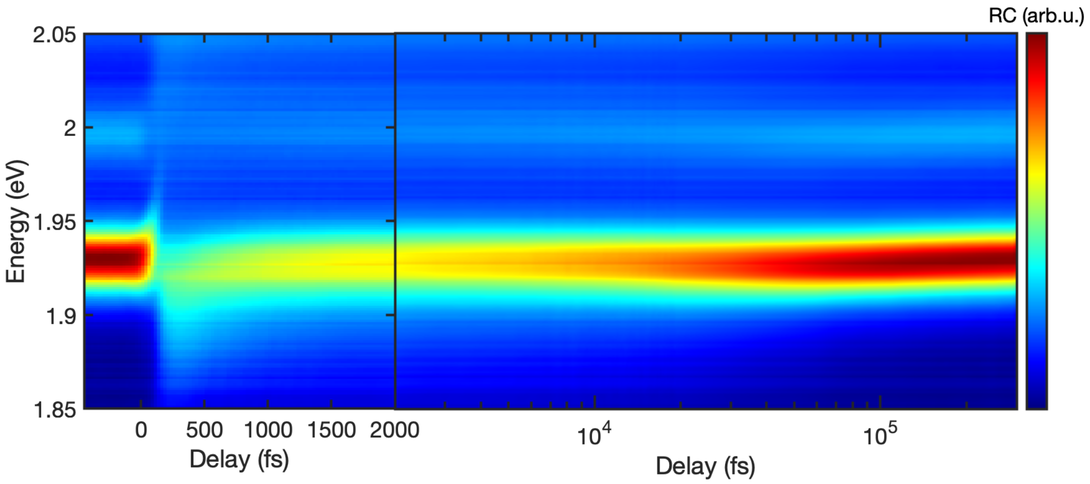

Supplementary Figure S5. Dynamic RC versus pump-probe time delay and probe photon energy for the MoS_2 BL out-of-cavity sample, excited with ∼30 $\mu J/cm^2$.

Figure S6a shows the transient peak amplitude variation of XA and hIX, which follows a double exponential decay. We fit the experimental exciton dynamics using a multi-exponential model, comprising a rise and two decay components, convoluted with the instrument response function of our setup [5]. We apply the same model to the transient polariton splittings (Fig. S6b), extracted from the data in Fig. 2c. In this case, we fitted the cavity data with two peaks even below the SC threshold, despite the large uncertainties. The table below summarizes the fitted values for the rise and decay times in the different cases, showing a very good match between the polariton splitting

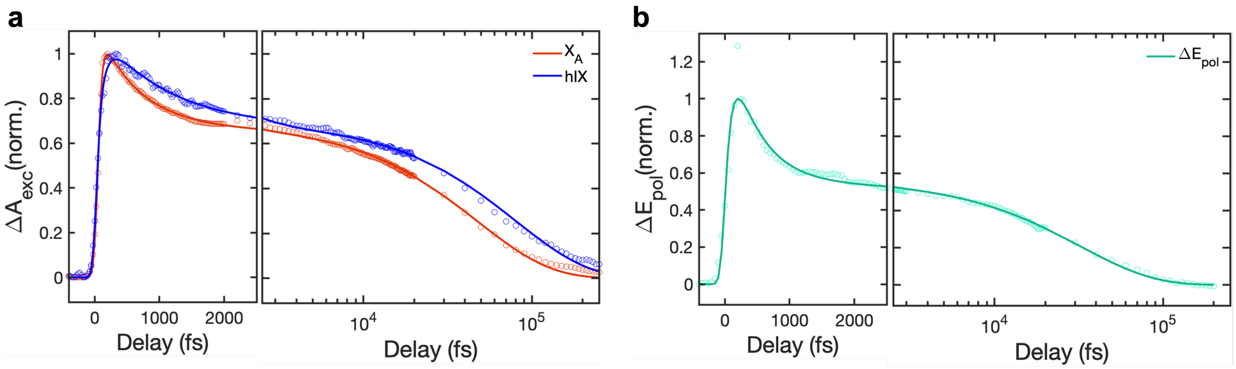

Supplementary Figure S6. a) Normalized exciton peak amplitude variation of X_A and hIX (red and blue dots respectively), extracted from the dynamic RC fits at different time delays. The experimental dynamics are fitted with multi-exponential curves (solid lines) in order to extract rise and decay times. b) Normalized polariton splitting as a function of pump-probe delay (green dots) extracted from Fig. 2d of the main text, fitted with a multi-exponential curve (solid line) to extract rise and decay times.

times and the X_A ones. Therefore, we conclude that SC collapse and recovery times are ruled by the optical saturation dynamics of the uncoupled excitons, or in other words by their formation and depopulation times.

	$\tau_{\rm rise}$ (fs)	τ_1 (fs)	τ_2 (ps)
X_A	60 ± 30	610 ± 40	47 ± 1
hIX	105 ± 20	1025 ± 80	80 ± 3
$\Delta E_{\rm pol}$	90 ± 30	455 ± 100	32 ± 3

SUPPLEMENTARY NOTE S6: SC SWITCHING IN ML CAVITIES

In this section, we discuss the results of the excitation of a MoS_2 ML cavity in SC regime with ultrafast pump pulses tuned at ≈ 1.94 eV, with an energy of 3.75 pJ (212.2 μJcm^{-2}). Figure S7a shows the relative transmittance spectra of the cavity probed as a function of the delay time between pump and probe pulses.

Similarly to the BL cavity case, we observed a strong modulation of the UPB and LPB energies due to optical saturation of excitons, reaching a full collapse into a weakly coupled cavity mode. This proves that exciton nonlinearities in MLs are also strong enough to allow a switching of the SC regime. However, differently from the BL cavity, the recovery of SC is much slower, failing to produce a full switching cycle within 1 ps. This is due to the slower depopulation mechanisms

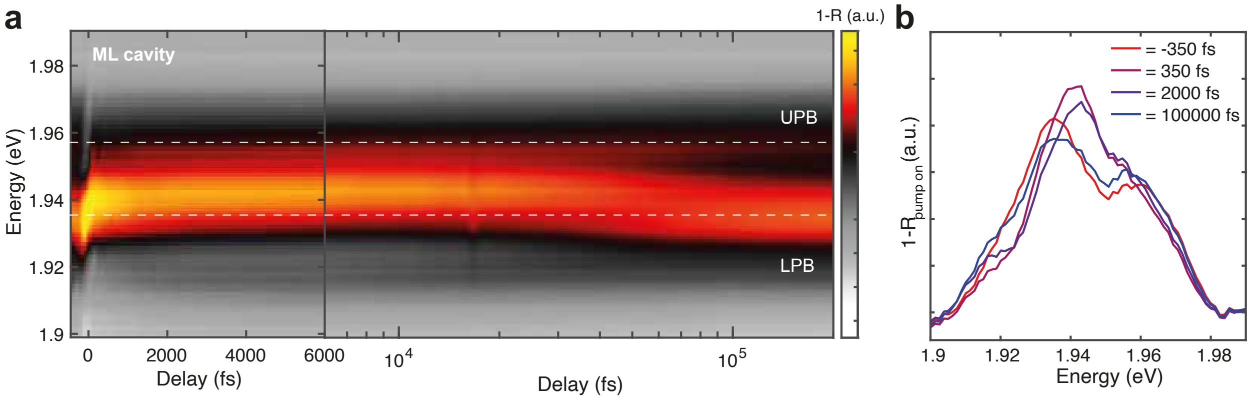

Supplementary Figure S7. a) Color map of the 1-R spectra of the ML microcavity as a function of the pump-probe delay showing the collapse and later revival of the UPB and LPB (white dashed lines). b) 1-R spectra of the ML microcavity taken at different pump-probe delays, extracted from panel (a).

for excitons in MLs, exhibiting overall a much slower decay compared to BLs, as discussed in Supplementary Note S1. We note that we used the same pump fluence of the experiments on the BL cavity shown in Fig.2 of the main text, which in that case led to an ultrafast complete switching cycle. On the other hand, decreasing the excitation fluence in the ML cavity to achieve the same result will not lead to a complete quenching of SC, but just a partial contraction of the Rabi splitting, due to an insufficiently high peak polariton density.

The on/off contrast in this case is worse than what achieved in the BL cavity, looking at the 1-R spectra at different delays in Fig. S7b, extracted from the map in the same figure. This is a consequence of the smaller Rabi splitting obtained in this system (see Supplementary Note S2), which is only slightly larger than the exciton linewidth, broadened by excitation-induced dephasing [6, 7].

SUPPLEMENTARY NOTE S7: TRANSFER MATRIX SIMULATIONS

In order to perform the simulations of the BL cavity optical response increasing the exciton density (Fig.3 of the main text) we used the Transfer Matrix Method (TMM) [8, 9]. With this theoretical approach, it is possible to calculate with high accuracy the reflectance, absorbance and transmittance of a multi-layer thin film optical structure, knowing the thicknesses and the complex refractive indexes of the layers. This method can be applied very well also to planar microcavities in SC regime [10]. For the layers of the DBR, PMMA spacer and silver mirrors, we used tabulated values of refractive index (http://refractiveindex.info). For the hBN we assumed a

constant refractive index of 1.85 [11], while we calculated the refractive index of the TMD fitting the experimental RC, using the Kramers-Kronig (KK) method [12, 13]. In particular, we used two Lorentzian functions to fit the excitonic optical response for X_{A-BL} and hIX, while we kept fixed the high frequency dielectric constant ϵ_∞ at 25 [14].

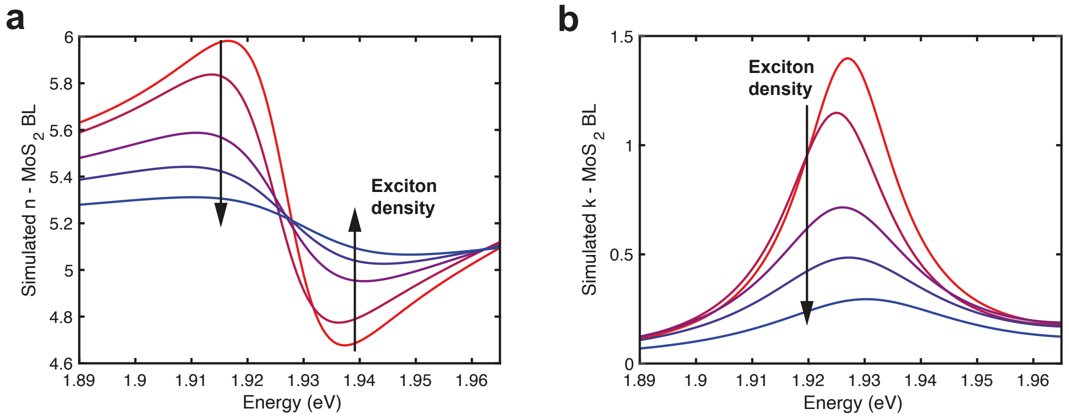

Supplementary Figure S8. a) Real part of the complex refractive index (n) of a MoS_2 BL calculated for an increasing exciton density, used in the TMM calculations of the microcavity optical response. b) Imaginary part of the complex refractive index (k) of a MoS_2 BL calculated for an increasing exciton density, used in the TMM calculations of the microcavity optical response.

To take into account the effects of the increased exciton density on the absorption bleaching, energy shift and spectral broadening of the exciton features, we fitted the experimental RC spectra of an out-of-cavity MoS_2 BL measured at different laser fluences in single narrowband ultrashort pulse experiments, reported in one of our previous works [15]. The real and imaginary part of the refractive index of the MoS_2 BL resulting from the KK analysis at different exciton densities are shown in Fig. S8. Using such density-dependent TMD optical constants, we found a good match between the experimental and calculated cavity 1-R spectra in Fig.3 of the main text, for increasing exciton densities of $1*10^4$, $2*10^4$, $3.5*10^4$, $5*10^4$ and $8*10^4$ μm^{-2}. We note that here we make the assumption of attributing the SC collapse mainly to the effect of the optical saturation of excitons, without taking into account other polaritonic effects in our simulations.

SUPPLEMENTARY NOTE S8: THEORY ON OPTICAL SATURATION IN MOS_2 BL CAVITIES

The spectrum of the probe field

In the experiment, the intralayer exciton (X_{A-BL}) in a bilayer MoS_2 is probed. The Hamiltonian of the probed exciton and microcavity coupled system can be written as

$$H = \begin{bmatrix} E_c - i\kappa & \frac{1}{2}g(n_X)\Omega_{A_{BL}} \\ \frac{1}{2}g(n_X)\Omega_{A_{BL}} & E_{A_{BL}} - i\gamma \end{bmatrix} \tag{S1}$$

where the E_c is the cavity-photon energy and $E_{A_{BL}}$ is the X_{A-BL} (probed) exciton energy. The cavity-photon and the probed exciton linewidth are κ and γ respectively. The nonlinear saturation in the arbitrary order can be written as

$$g(n_X) = e^{-\alpha n_X}, \tag{S2}$$

and it depends on the total density of exciton n_X in MoS_2. This includes bright and dark excitonic fractions, as saturation comes from Pauli statistical effects and relies on the presence of fermionic constituents. The factor $g(n_X)$ renormalizes the Rabi splitting such that coupling decreases in the presence of electrons and holes. The parameter α depends on the details of the exciton wavefunction. The estimation of α is outlined in Ref. [16].

The optical response of the cavity with bilayer MoS_2 embedded is determined by the polariton density of states $[\Xi(\omega)]$ which is given by the imaginary part of the photonic Green's function $\Xi(\omega) = \mathrm{Im}[G_c(\omega)]$. The full Green's function is

$$G(\omega) = [\omega - H]^{-1} = \begin{bmatrix} G_c(\omega) & D(\omega) \\ D^*(\omega) & G_X(\omega) \end{bmatrix} \tag{S3}$$

and the photonic part is the diagonal matrix element in $G(\omega)$ matrix located at the top left corner that we denote as $G_c(\omega)$. In this formalism, the hybridization of a polariton can be understood as the photon dressed by the excitons.

In the next section, we investigate $\Xi(\omega)$ under different pumping conditions. The formation of the excitonic occupation is determined by how the system is excited initially. This will lead to different time-dependent optical responses.

Time-evolution of the exciton density

To model the time-dependent total exciton density, we write the rate equation for the long-lived excitons (n_{R}) and the excitons created by pumping (n_{p}) as

$$\frac{dn_{\mathrm{p}}}{dt} = -\gamma_{\mathrm{p}} n_{\mathrm{p}} - r n_{\mathrm{p}} + \Theta(t), \tag{S4}$$

$$\frac{dn_{\mathrm{R}}}{dt} = -\gamma_{\mathrm{R}} n_{\mathrm{R}} + r n_{\mathrm{p}}, \tag{S5}$$

where γ_{p} is the pumped exciton decay rate, and r is the transfer rate from bright to dark excitons. For the pumping, we may let

$$\Theta(t) = \begin{cases} \Lambda \mathrm{e}^{-(\mathrm{t}-\mathrm{t}_0)^2/\mathrm{T}^2}, & 0 \leq t \\ 0, & \text{otherwise} \end{cases}, \tag{S6}$$

with Λ being the pumping rate. The length of the pulse is T and the peak of the pulse is at time t_0.

Using the boundary condition $n_{\mathrm{p,R}}(\infty) = 0$ and $n_{\mathrm{p,R}}(0) = 0$, we find the solution for the first order linear differential equation of n_{p} as

$$n_{\mathrm{p}}(t) = \mathrm{e}^{-(\gamma_{\mathrm{p}}+\mathrm{r})\mathrm{t}} \int_0^{\mathrm{t}} \mathrm{e}^{(\gamma_{\mathrm{p}}+\mathrm{r})\mathrm{t}'} \Theta(t') dt' \tag{S7}$$

Therefore, for the time evolution of the exciton population in the reservoir, we have

$$n_{\mathrm{R}}(t) = r\mathrm{e}^{-\gamma_{\mathrm{R}}\mathrm{t}} \int_0^{\mathrm{t}} \mathrm{e}^{\gamma_{\mathrm{R}}\mathrm{t}'} n_{\mathrm{p}}(t') dt' \tag{S8}$$

These give the total excitons population as

$$n_{\mathrm{X}}(t) = n_{\mathrm{p}}(t) + n_{\mathrm{R}}(t) \tag{S9}$$

where $n_{\mathrm{p}}(t)$ and $n_{\mathrm{R}}(t)$ can be easily numerically evaluated using Eqs. (S7) and (S8).

Comparison of the $\mathbf{X_{A-BL}}$ and hIX cavity pumping

In this section, we use Eqs.(S3), (S7), and (S8) to simulate the time-resolve spectrum in Figs. S9a - S9c for $\mathrm{X_{A-BL}}$ pumping and Fig.S9d for hIX pumping. In the $\mathrm{X_{A-BL}}$ pumping, we find a large portion of the pumped exciton transfer to the reservoir on the picosecond timescale [17]. This process is relevant only if the exciton lifetime is comparable to the transfer rate $r \approx \gamma_{\mathrm{p}}$. After all exciton transferring to the reservoir, the later nonlinear response is mostly determined by the

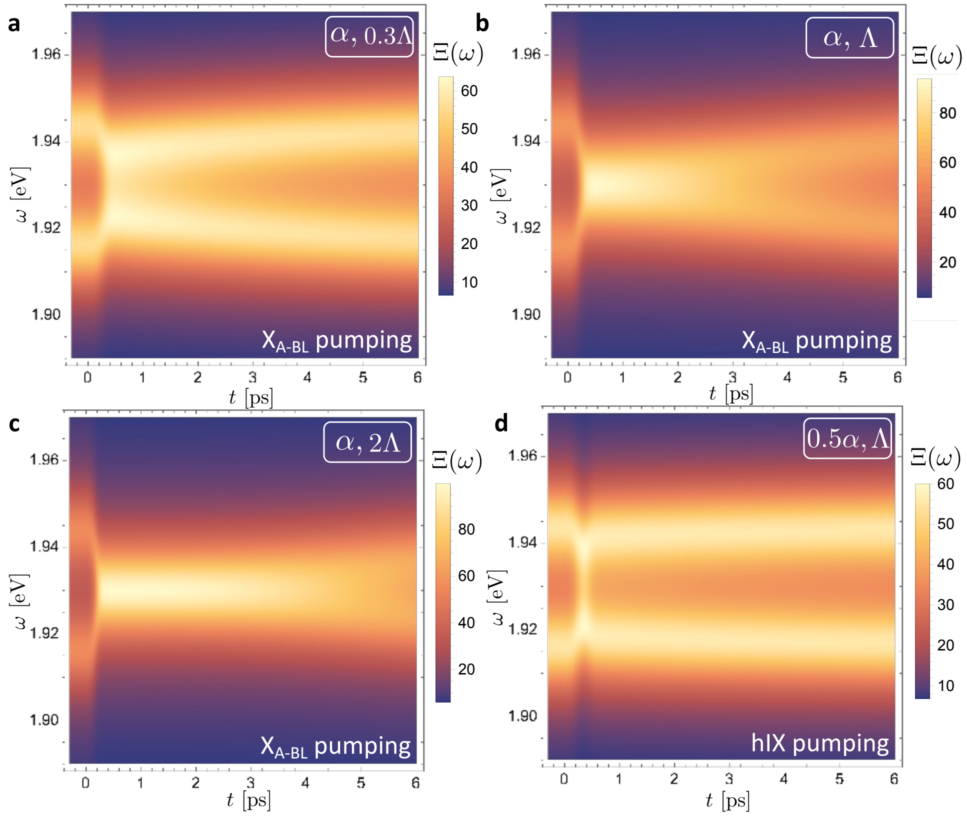

Supplementary Figure S9. a-c) Simulated pump fluence dependence of intralayer pumping. In this plot, we set $\gamma_\mathrm{p} = \gamma = 10\mathrm{meV}$, $r = \gamma_\mathrm{p}$, and $\gamma_\mathrm{R} = 0.01\gamma_\mathrm{p}$. The insets of the upper right corner show the relative strength of the saturation effects α and the pumping rate Λ. The nominal value of these quantities is set to $\alpha = 0.05L^2$ and $\Lambda = 0.1L^{-2}\mathrm{ps}^{-1}$ where L^2 being the size of the sample. The plots of $\Xi(\omega) = \mathrm{Im}[G_\mathrm{c}(\omega)]$ with pumping rate 0.3Λ in (a), Λ in (b), and 2Λ in (c) demonstrate the excitation fluence dependence of the time-resolved spectrum. d) hIX pumping. Most of the parameters are the same as in (**a**) - (**c**) except we use a slightly longer lifetime for hIX with $\gamma_\mathrm{p} = 8\mathrm{meV}$, smaller transferring rate $r = 0.1\gamma_\mathrm{p}$ to reservoir, and smaller saturation factor 0.5α.

long-lived state in the reservoir. This may explain the slower timescale in the SC recovery phase. In Fig. S9, we also simulate the excitation fluence dependence of the recovery time. The higher pump fluence leads to a longer recovery time since more long-lived excitons in the reservoir are created. Thus, the system requires a longer time to relax back to a lower exciton population to establish the SC phase. This trend is consistent with the experimental observation.

In contrast to the $\mathrm{X}_{\mathrm{A-BL}}$ pumping case, by pumping the hIX the transfer rate to the reservoir is slower ($r \approx 0.1\gamma_\mathrm{p}$). In such a case, a significant portion of the pumped hIX exciton recombines into photons before transferring into the reservoir. In the SC recovery phase, a much smaller long-lived exciton density remains in the reservoir. Additionally, the nonlinear saturation factor α is smaller

due to the mitigation of blockade effects (see main text Fig.4a) leading to a faster recovery time of SC, see Fig. S9d.

As we discussed in the main text, transferring a momentum-bright into momentum-dark states can be achieved by scattering with the impurities and phonon. These scattering may gives a smaller r in hIX. Particularly, hIX has weaker scattering with impurities due to the wavefunction spreading in the out-of-plane direction. Also, interacting with phonon, this can also lead to thermal relaxation that transfers the (high-energy) bright state and dark (low-energy) states. However, in a thermal process the rate r depends on the relative energies separation (Δ) between the high-energy state and low-energy states. The smaller of the energy separation results in smaller r, since thermal fluctuations allow the opposite transition from low-energy to high-energy states. The transition amplitude of this adverse effects is roughly given by the Boltzmann factor $\mathrm{e}^{-\Delta/(\mathrm{k_B T})}$. In small Δ case, the low-to-high energy transition may not be strongly suppressed and reverse the relaxation process in converting the bright states to the reservoir leading to a smaller r. As suggested in Ref. [18], there exist many low energy momentum-dark states for hIX which are potentially close to the bright hIX. The transition between these states may has a smaller r. However, to be more conclusive, we note that the transition between these states in Ref. [18] due to the scattering with impurities and phonon required further detailed investigations.

SUPPLEMENTARY NOTE S9: DOUBLE BILAYER MICROCAVITY

To form a double BL cavity two bilayers were overlapped on the bottom DBR to make a double bilayer structure with a separation of 40 nm of hBN between them. The cavity was again completed by a PMMA spacer and silver mirror with the same thicknesses used for the single BL cavity. We measure the cavity reflectivity spectra as a function of the angle and for each angle we fit the spectrum with Lorentzians. The extracted peak energies are then fitted with a three-level coupled oscillators model, such that

$$H_{\mathrm{DBL}} = \begin{pmatrix} E_{\mathrm{c}} & \Omega_{\mathrm{A_{DBL}}} & \Omega_{\mathrm{hIX_{DBL}}} \\ \Omega_{\mathrm{A_{DBL}}} & E_{\mathrm{A_{DBL}}} & 0 \\ \Omega_{\mathrm{hIX_{DBL}}} & 0 & E_{\mathrm{hIX_{DBL}}} \end{pmatrix}$$

As can be seen in Fig S10, two anticrossings can be observed at the energies of $\mathrm{X_A}$ and hIX. The latter are at 1.932 and 1.992 eV for $\mathrm{A_{DBL}}$ and $\mathrm{hIX_{DBL}}$, respectively, with a slight redshift of both excitons by 8 meV, with the intra to interlayer exciton energy separation being consistent with the single bilayer case. We attribute such small exciton redshift to the different dielectric

environment experienced by the excitons compared to the single BL case, due to the presence of thick hBN between the BLs. In fact, it has been shown that changing the dielectric environment can shift the exciton energy up to tens of meVs [19]. The cavity has a negative detuning of 11 meV from A_{DBL}. The corresponding Rabi splittings are extracted as $\Omega_{A_{DBL}} = 55.0 \pm 0.5$ meV and $\Omega_{hIX_{DBL}} = 31.8 \pm 0.9$ meV.

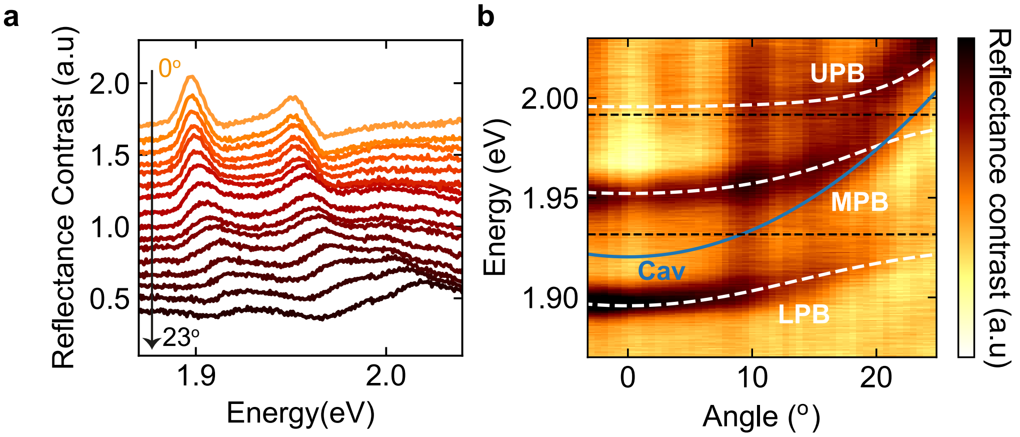

Supplementary Figure S10. a) Waterfall plot of the RC spectra as a function of angle for a double bilayer MoS_2 embedded in a microcavity. Two anticrossings are observed at the X_A and hIX energies, respectively. b) Energy-angle map of the cavity reflectivity spectra. The fitted Upper, Middle and Lower polariton branches (UPB, MPB, LPB) are shown as white dashed curves. The cavity mode (Cav) is shown as a blue solid curve, whereas the exciton energies are shown as black dashed lines.

SUPPLEMENTARY NOTE S10: ROOM TEMPERATURE SC SWITCHING

To better compare our approach with systems working at RT, we have demonstrated SC switching with a newly fabricated cavity with a single BL MoS_2 working at ambient conditions. As shown in Fig.S11, owing to the collapse of SC regime, the LP can be shifted by about 20 meV, using a very low pump pulse energy of $\sim$1.8 pJ.

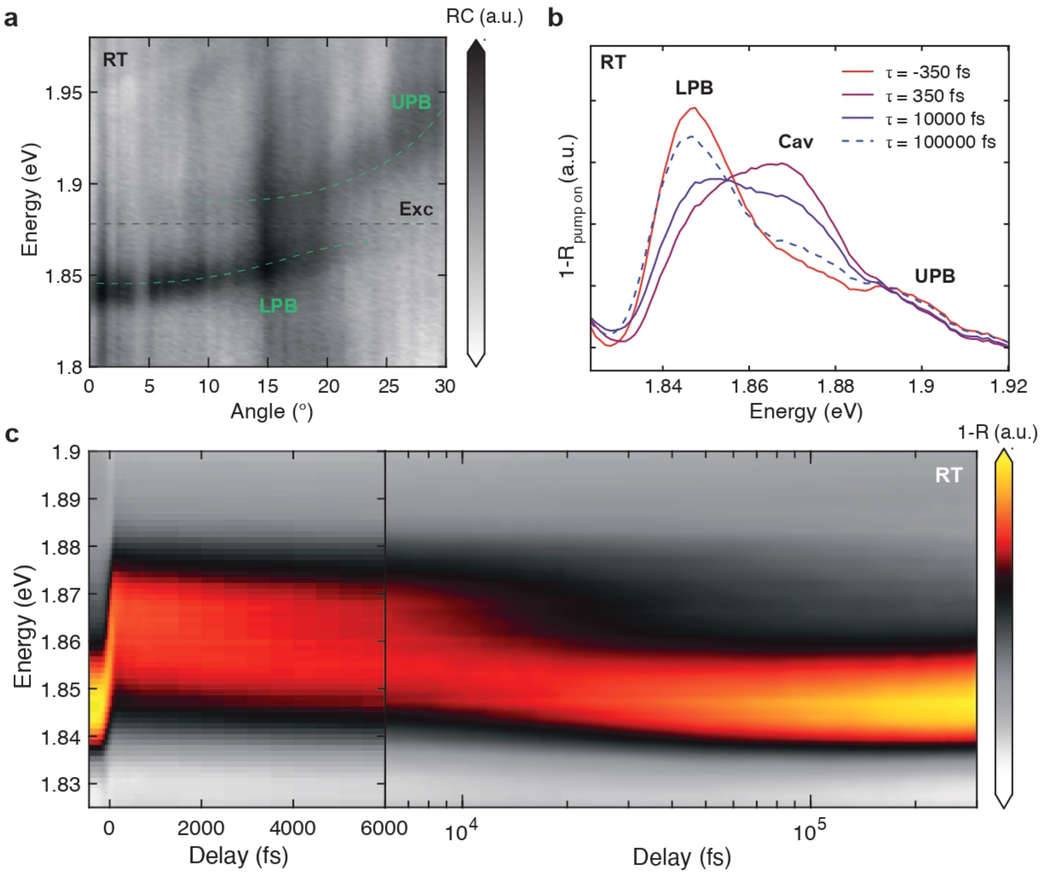

Supplementary Figure S11. a) BL MoS_2 microcavity dispersion measured at RT showing a clear anticrossing of upper and lower polariton branches (dashed green lines are guides for the eye) around the exciton energy (dashed black line). b) 1-R spectra of the RT cavity taken at different pump-probe delays. c) Color map of the 1-R spectra versus pump-probe delay time of a single MoS_2 BL microcavity excited at RT with pump pulses tuned at 1.91 eV.

SUPPLEMENTARY NOTE S11: POLARITON LINEWIDTHS TEMPORAL DYNAMICS

In this section we discuss the trasient behaviour of the polariton linewidths after the SC collapse. Immediately after the SC switching, when the Rabi splitting is still partially quenched, the polariton linewidths show an additional broadening due to excitation induced dephasing on the excitonic resonances [7]. After few tens of ps, when the exciton/polariton density decreases, a narrowing occurs since such effects are fading away. Fig.S12 displays the polariton peak position and linewidth (depicted by the shaded areas) for different delay times up to 200 ps, extracted from Gaussian fits of the reflectivity data of Fig.2 of the main text.

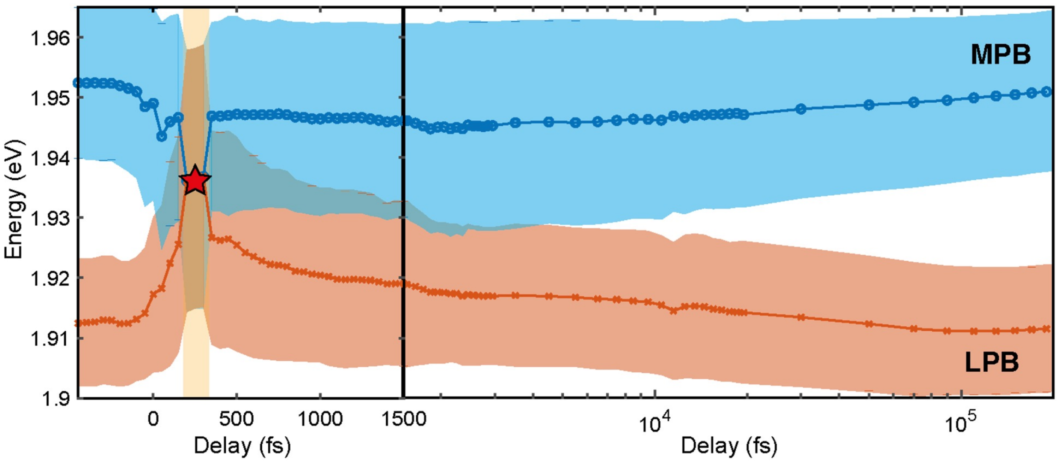

Supplementary Figure S12. Results of Gaussian fits of the polariton/cavity modes dynamic spectra extracted from Fig. 2d of the main text, shown up to 200 ps. The blue (orange) trace refers to the MPB (LPB) peak energy, while the shaded areas depict the linewidth of the polariton modes (Full Width Half Maximum, FWHM).

SUPPLEMENTARY NOTE S12: EFFECTS OF THE DETUNING ON THE SC SWITCHING

We tested the SC switching in a single BL cavity with a slightly negative detuning, as shown in Fig.S13. Before the pump excitation, the negative detuning makes the LPB more visible than MPB, while the weakly coupled cavity mode, visible after the collapse of SC, is redshifted compared to the sample shown in Fig.2 of the main text. Comparing the effective on/off extinction ratio calculated for this sample (Fig.S13c) to the one of the single BL cavity presented in Fig.5 of the main text, we observe an increase of the maximum absolute contrast ratio, aided by the more negative detuning, reaching similar values of the double BL sample. However, the high contrast spectral window is localized only around 1.93 eV, between the LPB and MPB. A double BL cavity instead ensures high contrast in a broader energy range, i.e. also in the spectral region of the LPB. This is due to the increased energy separation between LPB and the cavity mode in the double BL sample due to the higher Rabi splitting. In principle, further increasing the Rabi splitting, adding more BL stacks for example, would allow to reduce even more the polariton linewidth, reaching the exciton homogeneous linewidth limit, as recently proposed [20]. This will also increase the on/off extinction ratio in an ultrafast SC switching experiment.

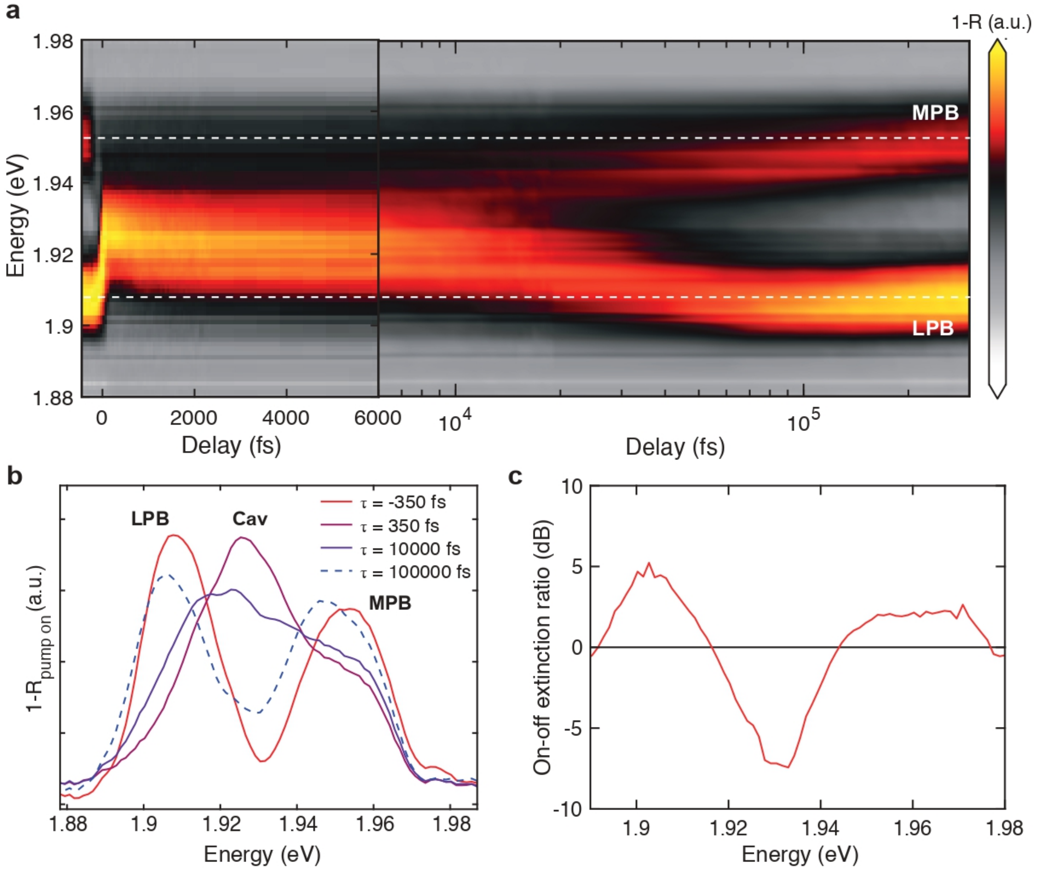

Supplementary Figure S13. a) Color map of the 1-R spectra versus pump-probe delay time of a negatively detuned single MoS_2 BL microcavity excited with pump pulses at 1.91 eV. b) 1-R spectra extracted as vertical cross-sections from the map in a) at different pump-probe delays. c) Effective on/off extinction ratio calculated from the 1-R spectra taken at -350 fs and 350 fs for the negatively detuned single BL cavity.

SUPPLEMENTARY NOTE S13: COMPARISON WITH PREVIOUS WORKS ON DYNAMIC SC MODULATION

Optical pump-probe spectroscopy has been previously used to study polariton ultrafast dynamics for different classes of materials strongly coupled to optical resonators. Here we provide a detailed comparison of our results with previous studies on transient SC modulation, divided into different subsections, one for each class of materials.

Quantum wells

Early works on semiconductor Quantum wells (QWs) in planar microcavities show the transition from strong to weak coupling regime due to phase space filling effects increasing the pump power [21], also monitoring the cavity behavior in time [22], but without presenting a time evolution of the strong-to-weak coupling switching. More recently, the ultrafast (a few ps) collapse of SC in QWs was dynamically tracked in a pump-probe experiment, but the SC recovery time was not reported, falling outside the time window of study (20 ps) [23]. In general, the long lifetime of excitons in QWs hinders a fast polariton recovery after the SC breakdown. Multiple QWs systems also support intersubband transitions in the THz range which have been used to demonstrate an ultrafast switch-on of the SC after photoexcitation of electrons in the first subband, happening in few tens of fs [24]. A similar active layer has been employed in intersubband polaritonic metasurfaces to demonstrate highly nonlinear optical saturation and all-optical modulation of the SC operating in the THz range, reaching eventually a complete collapse of the polariton splitting. In this system a fast modulation time of about 2 ps was reported, but only exciting the sample far below the strong to weak coupling transition power threshold [25].

Organic semiconductors

Pump-probe experiments on organic microcavities in SC regime have also been previously reported, showing for example the ultrafast transfer of population between polariton states [26] occurring even over long distances (in the micron scale) [27], or coherent polariton oscillations due to quantum beating between the coupled states [28, 29]. It has been reported that organic polaritons in microcavities can show a Rabi contraction, i.e. a small decrease in the Rabi splitting, when excited with high power femtosecond laser pulses [30]. Signatures of a full collapse of SC using organic semiconductors have been observed only in plasmonic systems, probably due to the large local EM field enhancement close to the nanostructures, at 77 K [31] and room temperature [32], although with very poor switching contrast. Moreover, the pump pulse energies used in these experiments are in the order of tens of nJ, close to the threshold of photo-induced chemical modifications or degradation of the materials.

2D semiconductors

Only very few works have been published on pump-probe experiments on strongly coupled TMD systems. Upper and lower polariton states in a TMD microcavity can exhibit a polarization-dependent ultrafast blueshift of hundreds of µeVs in one valley of the monolayer, pumping below the polariton energies with a circularly polarized ultrashort pulse, due to valley-selective exciton Stark shift [2]. More recently, Tang et al. showed that an ultrafast modulation of the Rabi splitting of few meVs can be achieved in TMD monolayers transferred on top of plasmonic nanodisks, but without achieving a complete SC collapse [7]. The SC collapse has been demonstrated in static experiments on TMD monolayers and bilayers acting on different excitonic species, i.e. neutral excitons, trions, Rydberg excitons or hybridized interlayer excitons [15, 33–37], or using a stack of TMD monolayers separated by hBN [38], illuminating them with single high energy laser pulses. Nevertheless, the latter works neither provided a study on the strong to weak coupling switching dynamics nor showed its application for ultrafast all-optical switching

* Contributed equally

† a.tartakovskii@sheffield.ac.uk

‡ giulio.cerullo@polimi.it

[1] P. D. Cunningham, A. T. Hanbicki, T. L. Reinecke, K. M. McCreary, and B. T. Jonker, Resonant optical stark effect in monolayer ws2, Nature communications **10**, 5539 (2019).

[2] T. LaMountain, J. Nelson, E. J. Lenferink, S. H. Amsterdam, A. A. Murthy, H. Zeng, T. J. Marks, V. P. Dravid, M. C. Hersam, and N. P. Stern, Valley-selective optical stark effect of exciton-polaritons in a monolayer semiconductor, Nature communications **12**, 4530 (2021).

[3] Z. Nie, R. Long, L. Sun, C.-C. Huang, J. Zhang, Q. Xiong, D. W. Hewak, Z. Shen, O. V. Prezhdo, and Z.-H. Loh, Ultrafast carrier thermalization and cooling dynamics in few-layer mos2, ACS nano **8**, 10931 (2014).

[4] N. U. Din, V. Turkowski, and T. S. Rahman, Ultrafast charge dynamics and photoluminescence in bilayer mos2, 2D Materials **8**, 025018 (2021).

[5] C. Trovatello, F. Katsch, N. J. Borys, M. Selig, K. Yao, R. Borrego-Varillas, F. Scotognella, I. Kriegel, A. Yan, A. Zettl, et al., The ultrafast onset of exciton formation in 2d semiconductors, Nature communications **11**, 5277 (2020).

[6] F. Katsch, M. Selig, and A. Knorr, Exciton-scattering-induced dephasing in two-dimensional semiconductors, Physical Review Letters **124**, 257402 (2020).

[7] Y. Tang, Y. Zhang, Q. Liu, K. Wei, X. Cheng, L. Shi, and T. Jiang, Interacting plexcitons for designed ultrafast optical nonlinearity in a monolayer semiconductor, Light: Science & Applications **11**, 94 (2022).

[8] S. J. Byrnes, Multilayer optical calculations, arXiv preprint arXiv:1603.02720 (2016).

[9] S. Calati, Q. Li, X. Zhu, and J. Stähler, Ultrafast evolution of the complex dielectric function of monolayer ws 2 after photoexcitation, Physical Chemistry Chemical Physics **23**, 22640 (2021).

[10] D. J. Gillard, A. Genco, S. Ahn, T. P. Lyons, K. Yeol Ma, A. R. Jang, T. Severs Millard, A. A. Trichet, R. Jayaprakash, K. Georgiou, D. G. Lidzey, J. M. Smith, H. Suk Shin, and A. I. Tartakovskii, Strong exciton-photon coupling in large area $MoSe_2$ and WSe_2 heterostructures fabricated from two-dimensional materials grown by chemical vapor deposition, 2D Materials **8** (2021).

[11] D. Golla, K. Chattrakun, K. Watanabe, T. Taniguchi, B. J. LeRoy, and A. Sandhu, Optical thickness determination of hexagonal boron nitride flakes, Applied Physics Letters **102** (2013).

[12] A. Kuzmenko, Kramers–kronig constrained variational analysis of optical spectra, Review of scientific instruments **76** (2005).

[13] Y. Li, A. Chernikov, X. Zhang, A. Rigosi, H. M. Hill, A. M. Van Der Zande, D. A. Chenet, E.-M. Shih, J. Hone, and T. F. Heinz, Measurement of the optical dielectric function of monolayer transition-metal dichalcogenides: Mos 2, mo s e 2, ws 2, and ws e 2, Physical Review B **90**, 205422 (2014).

[14] F. Huang, Optical contrast of atomically thin films, The Journal of Physical Chemistry C **123**, 7440 (2019).

[15] C. Louca, A. Genco, S. Chiavazzo, T. P. Lyons, S. Randerson, C. Trovatello, P. Claronino, R. Jayaprakash, X. Hu, J. Howarth, et al., Interspecies exciton interactions lead to enhanced nonlinearity of dipolar excitons and polaritons in mos2 homobilayers, Nature Communications **14**, 3818 (2023).

[16] K. W. Song, S. Chiavazzo, and O. Kyriienko, Microscopic theory of nonlinear phase space filling in polaritonic lattices, Physical Review Research **6**, 023033 (2024).

[17] M. Selig, G. Berghäuser, A. Raja, P. Nagler, C. Schüller, T. F. Heinz, T. Korn, A. Chernikov, E. Malic, and A. Knorr, Excitonic linewidth and coherence lifetime in monolayer transition metal dichalcogenides, Nature Communications **7**, 13279 (2016).

[18] T. Deilmann and K. S. Thygesen, Finite-momentum exciton landscape in mono- and bilayer transition metal dichalcogenides, 2D Materials **6**, 035003 (2019).

[19] S. Borghardt, J.-S. Tu, F. Winkler, J. Schubert, W. Zander, K. Leosson, and B. E. Kardynał, Engineering of optical and electronic band gaps in transition metal dichalcogenide monolayers through external dielectric screening, Physical review materials **1**, 054001 (2017).

[20] X. Li, H. Wang, Y. Zhou, S. Luo, H. Zhou, Y. Zhu, H. Zhang, L. Zhang, and Z. Chen, Van der waals exciton polaritons with linewidth approaching homogeneous limit, Physical Review Materials **8**, L101002 (2024).

[21] R. Butté, G. Delalleau, A. Tartakovskii, M. Skolnick, V. Astratov, J. Baumberg, G. Malpuech, A. Di Carlo, A. Kavokin, and J. Roberts, Transition from strong to weak coupling and the onset of lasing in semiconductor microcavities, Physical Review B **65**, 205310 (2002).

[22] S. Jiang, S. Machida, Y. Takiguchi, Y. Yamamoto, and H. Cao, Direct time-domain observation of transition from strong to weak coupling in a semiconductor microcavity, Applied physics letters **73**, 3031 (1998).

[23] N. Takemura, M. D. Anderson, S. Trebaol, S. Biswas, D. Oberli, M. T. Portella-Oberli, and B. Deveaud, Dephasing effects on coherent exciton-polaritons and the breakdown of the strong coupling regime, Physical Review B **92**, 235305 (2015).

[24] G. Günter, A. A. Anappara, J. Hees, A. Sell, G. Biasiol, L. Sorba, S. De Liberato, C. Ciuti, A. Tredicucci, A. Leitenstorfer, et al., Sub-cycle switch-on of ultrastrong light–matter interaction, Nature **458**, 178 (2009).

[25] S. A. Mann, N. Nookala, S. C. Johnson, M. Cotrufo, A. Mekawy, J. F. Klem, I. Brener, M. B. Raschke, A. Alù, and M. A. Belkin, Ultrafast optical switching and power limiting in intersubband polaritonic metasurfaces, Optica **8**, 606 (2021).

[26] T. Virgili, D. Coles, A. Adawi, C. Clark, P. Michetti, S. Rajendran, D. Brida, D. Polli, G. Cerullo, and D. Lidzey, Ultrafast polariton relaxation dynamics in an organic semiconductor microcavity, Physical Review B—Condensed Matter and Materials Physics **83**, 245309 (2011).

[27] M. Russo, K. Georgiou, A. Genco, S. De Liberato, G. Cerullo, D. G. Lidzey, A. Othonos, M. Maiuri, and T. Virgili, Direct evidence of ultrafast energy delocalization between optically hybridized j-aggregates in a strongly coupled microcavity, Advanced Optical Materials **12**, 2400821 (2024).

[28] P. Vasa, W. Wang, R. Pomraenke, M. Lammers, M. Maiuri, C. Manzoni, G. Cerullo, and C. Lienau, Real-time observation of ultrafast rabi oscillations between excitons and plasmons in metal nanostructures with j-aggregates, Nature Photonics **7**, 128 (2013).

[29] D. Timmer, M. Gittinger, T. Quenzel, S. Stephan, Y. Zhang, M. F. Schumacher, A. Lützen, M. Silies, S. Tretiak, J.-H. Zhong, et al., Plasmon mediated coherent population oscillations in molecular aggregates, Nature Communications **14**, 8035 (2023).

[30] C. A. DelPo, B. Kudisch, K. H. Park, S.-U.-Z. Khan, F. Fassioli, D. Fausti, B. P. Rand, and G. D. Scholes, Polariton transitions in femtosecond transient absorption studies of ultrastrong light–molecule coupling, The journal of physical chemistry letters **11**, 2667 (2020).

[31] P. Vasa, R. Pomraenke, G. Cirmi, E. De Re, W. Wang, S. Schwieger, D. Leipold, E. Runge, G. Cerullo, and C. Lienau, Ultrafast manipulation of strong coupling in metal- molecular aggregate hybrid nanostructures, Acs Nano **4**, 7559 (2010).

[32] J. Kuttruff, M. Romanelli, E. Pedrueza-Villalmanzo, J. Allerbeck, J. Fregoni, V. Saavedra-Becerril, J. Andréasson, D. Brida, A. Dmitriev, S. Corni, et al., Sub-picosecond collapse of molecular polaritons to pure molecular transition in plasmonic photoswitch-nanoantennas, Nature Communications **14**, 3875 (2023).

[33] R. Emmanuele, M. Sich, O. Kyriienko, V. Shahnazaryan, F. Withers, A. Catanzaro, P. Walker, F. Benimetskiy, M. Skolnick, A. Tartakovskii, et al., Highly nonlinear trion-polaritons in a monolayer semiconductor, Nature communications **11**, 3589 (2020).

[34] T. Lyons, D. Gillard, C. Leblanc, J. Puebla, D. Solnyshkov, L. Klompmaker, I. Akimov, C. Louca, P. Muduli, A. Genco, et al., Giant effective zeeman splitting in a monolayer semiconductor realized by spin-selective strong light–matter coupling, Nature Photonics **16**, 632 (2022).

[35] J. Gu, V. Walther, L. Waldecker, D. Rhodes, A. Raja, J. C. Hone, T. F. Heinz, S. Kéna-Cohen, T. Pohl, and V. M. Menon, Enhanced nonlinear interaction of polaritons via excitonic rydberg states in monolayer wse2, Nature communications **12**, 2269 (2021).

[36] L. Zhang, F. Wu, S. Hou, Z. Zhang, Y.-H. Chou, K. Watanabe, T. Taniguchi, S. R. Forrest, and H. Deng, Van der waals heterostructure polaritons with moiré-induced nonlinearity, Nature **591**, 61 (2021).

[37] B. Datta, M. Khatoniar, P. Deshmukh, F. Thouin, R. Bushati, S. De Liberato, S. K. Cohen, and V. M. Menon, Highly nonlinear dipolar exciton-polaritons in bilayer mos2, Nature communications **13**, 6341 (2022).

[38] J. Zhao, A. Fieramosca, K. Dini, R. Bao, W. Du, R. Su, Y. Luo, W. Zhao, D. Sanvitto, T. C. Liew, et al., Exciton polariton interactions in van der waals superlattices at room temperature, Nature Communications **14**, 1512 (2023).
